# OPTIMIZING STAFF RECRUITMENT AND RETENTION IN CARE HOMES: WHAT WORKS? THE REACH REALIST REVIEW

**DOI:** 10.1093/geroni/igae098.1949

**Published:** 2024-12-31

**Authors:** Reena Devi, Kirsty Haunch, Karen Spilsbury, Sonia Dalkin, Angela Bate, Liz Jones, Claire Goodman, Natalie King

**Affiliations:** University of Leeds, Leeds, England, United Kingdom; University of Leeds, Leeds, England, United Kingdom; University of Leeds, Leeds, England, United Kingdom; Northumbria University Newcastle, Newcastle, England, United Kingdom; Northumbria University Newcastle, Newcastle, England, United Kingdom; National Care Forum, Nottingham, England, United Kingdom; University of Hertfordshire, Hertfordshire, England, United Kingdom; University of Leeds, Leeds, England, United Kingdom

## Abstract

Solutions for sustaining the social care workforce need to a) consider the different factors which influence staff experiences, b) recognize the nuances between different care settings, and c) differentiate between staff groups (e.g. age, levels of experience). At the University of Leeds, we led research which uses a realist synthesis approach to reviewing international academic and grey literature and contributions from sector stakeholders to develop theories. The results identified strategies used in care homes to attract, recruit, and retain Registered Nurses and care workers, e.g., effective job interviews, opportunities for career development, rewards and recognition, flexible working options, and caring conversations. The strategies used to attract, recruit, and retain staff do not operate independently, they interact and work together. We brought the evidence-based programme theories together into an explanatory framework to explain how and why these strategies work, for which staff, the conditions needed for strategies to work, and the outcomes to be expected. The framework summarizes the different ways the strategies help with attracting, recruiting and retaining staff. For example, empowering staff through caring conversations and opportunities for career development; building and fulfilling a psychological contract to set accurate expectations of care work; and reciprocating positive experiences (when providing staff with positive experiences staff will reciprocate and respond positively (i.e. commitment and loyalty at work). We will summarize the full findings during the symposium. To the best of our knowledge, this is the first time a realist synthesis approach has been used to review the literature in this area.

